# A need to improve the assessment of environmental hazards for falls on stairs and in bathrooms: results of a scoping review

**DOI:** 10.1186/s12877-018-0958-1

**Published:** 2018-11-09

**Authors:** Rosanne Blanchet, Nancy Edwards

**Affiliations:** 1 School of Nursing, University of Ottawa, 1 Stewart Street, Room 212, Ottawa, ON K1H 8M5 Canada; 2 School of Nursing, University of Ottawa, 1 Stewart Street, Room 205, Ottawa, ON K1H 8M5 Canada

**Keywords:** Environmental hazards, Falls, Seniors, Built environment, Scoping review, Bathroom, Stairs

## Abstract

**Background:**

Falls occurring on stairs or in bathrooms are associated with a high risk of injuries among older adults. Home environmental assessments are frequently used to guide fall-prevention interventions. The aims of this review were to describe how, where, by whom, and for whom environmental hazard checklists are used, and to examine the characteristics of environmental hazard assessment checklists with specific attention to features of bathrooms and stairs/steps assessed in them.

**Methods:**

Studies published before January 5, 2018, were identified using several databases. Publications reporting the use and/or evaluation of environmental hazard checklists were eligible if they assessed bathrooms or stairs/steps in homes of older adults (≥65 years). Content analysis was conducted on publications that provided a complete list of specific environmental hazards assessed. Checklist items related to bathrooms and stairs/steps were extracted and categorized as structural or non-structural and as objective or subjective.

**Results:**

1119 studies were appraised. A pool of 136 published articles and 4 checklists from the grey literature were included in this scoping review. Content analysis was conducted on 42 unique checklists. There was no widely used checklist and no obvious consensus definition of either environmental hazards overall or of single hazards listed in checklists. Checklists varied greatly with respect to what rooms were assessed, whether or not outdoor stair/steps hazards were assessed, and how responses were coded. Few checklists examined person-environment fit. The majority of checklists were not oriented towards structural hazards in bathrooms. Although the majority of checklists assessing stair/steps hazards evaluated structural hazards, most features assessed were not related to the construction geometry of stairs/steps. Objective features of bathrooms and stairs/steps that would deem them safe were rarely specified. Rather, adequacy of their characteristics was mostly subjectively determined by the evaluator with little or no guidance or training.

**Conclusion:**

The lack of standard definitions and objective criteria for assessing environmental hazards for falls is limiting meaningful cross-study comparisons and slowing advances in this field. To inform population health interventions aimed at preventing falls, such as building code regulations or municipal housing by-laws, it is essential to include objectively-assessed structural hazards in environmental checklists.

**Electronic supplementary material:**

The online version of this article (10.1186/s12877-018-0958-1) contains supplementary material, which is available to authorized users.

## Background

Falls among older adults are considered a major public health concern [1]. Falls can lead to loss of autonomy, greater isolation and depression, reduced mobility, and increased morbidity and mortality [2]. In Canada, the direct and indirect costs of falls among older adults are estimated at over $3 billion annually [3]. Aging-in-place policies highlight the importance of mitigating fall risks in the home [4]; safer homes may enable independent rather than dependent living arrangements for older persons.

Although causes of falls are considered multi-factorial, it is well-established that environmental hazards are implicated in as many as one third of all falls among older adults [5–9]. Research on falls indicates that two areas in the home are particularly hazardous for injurious falls; bathrooms, and indoor or outdoor stairs or steps [10–12]. In the most recently available National Electronic Injury Surveillance data for 2017, for example, the product category stairs, ramps, landings and floors is the top-ranked location of injuries in the United States for those 65 years and older, while bathtub and shower structures rank fourth for this age group [13]. Furthermore, when time spent on stairs or in bathrooms (risk exposure time) is taken into account, these locations account for a significantly higher incidence of falls than other room locations (Jake Pauls, personal communications, June 12, 2018). Stairs and bathrooms are problematic because they involve navigating transitions and transfers, and structural features of these locations (such as poor stair geometry or the lack of transfer assists) may challenge an individual’s capacity to respond to the pressure exerted by these environmental features, thereby exceeding optimal person-environment fit parameters [14–23].

Both primary studies and systematic reviews have documented the effectiveness of efforts to address environmental hazards generally, or more specifically in bathrooms and stairs [4, 6, 24–30]. Still, studies that assessed the influence of home environmental hazards, or of removing such hazards, on the occurrence of falls have frequently shown no significant associations [5, 31–39] or conflicting results [7] even if this relationship makes intuitive sense. It is our contention that these discrepant findings are influenced by how and which hazards are assessed or removed. Indeed, systematic reviews of fall prevention initiatives show that a variety of checklists have been used to assess environmental hazards and that information about their strengths and weaknesses is sparse [4, 6, 40]. Therefore, a review of what environmental hazard checklists have been developed and used is needed to more effectively prevent falls and to assess the potential for data on environmental hazards to inform policies such as building code legislation and regulated universal design.

The purpose of this scoping review was three-fold: a) to summarise how environmental hazards are defined by those developing or using environmental hazard checklists; b) to describe how, where, by whom, and for whom environmental hazard checklists are used; and, c) to examine the characteristics of environmental hazard checklists, with specific attention to features of bathrooms, and stairs/steps assessed in same. This review complements those that have focused on the relationships between falls and environmental hazards [4, 6, 40, 41] and provides a detailed examination of the assessment criteria used for two important locations in homes for injurious falls involving environmental hazards, namely bathrooms and stairs/steps.

## Methods

This scoping review was conducted in a systemic manner according to the steps outlined by Arksey and O’Malley [42], and Levac et al. [43]. Reporting follows the PRISMA (Preferred Reporting Items for Systematic Reviews and Meta-Analyses) statement guidelines, as appropriate. Ethics approval was not required.

### Identification of relevant articles

Papers were identified using various databases, namely: Medline, Embase, Web of Science, Scopus, CINAHL, AgeLine, HAPI, and PsychTESTS. No restrictions were set regarding the publication year. The search covered articles published up to January 5, 2018. A combination of descriptors (e.g. MESH terms) and key words was used. The authors reviewed the search syntax and strategy and provided additional search terms. The search strategy was finalized after consultation with a professional librarian and tailored for each database (Additional file 1). As an example, the following strategy was used for the search in Medline:

- (Fall OR accident OR accidental fall)
- AND (home adj3 hazard* OR environment* adj3 hazard*)
- AND (housing OR public housing OR Housing for the elderly OR home OR dwelling)

Backward searching from reference lists of reviewed articles was also done.

#### Inclusion and exclusion criteria

We applied inclusion and exclusion criteria in two stages. The first stage yielded a more complete set of articles, all with at least some information about environmental hazard checklists. For the first stage, the inclusion criteria were:

- Assess environmental hazards for falls in one or more of the following settings: personal homes or apartments, public housing, and housing for older persons including retirement residences, even if the checklist was not entirely described.
- Include an assessment of environmental hazards in bathrooms and/or on stairs/steps by lay and/or professional raters (e.g. nurses providing home healthcare services).
- Involve a population aged 65 years of age or older.
- Primary research study or research protocols for primary studies.

Exclusion criteria used for this first stage were:

- Focus exclusively on hospital or long-term care settings.
- Focus exclusively on a population aged less than 65 years of age (e.g. children).
- Not written in English or French.
- Conference and poster abstracts; letters, commentaries, editorials, reviews (e.g. narrative reviews, systematic reviews, meta-analysis studies), and practice guideline papers.

The second stage identified a subset of publications included in stage one that either included the checklists or provided a list of all specific environmental hazards assessed.

### Study selection

**Fig. 1:**
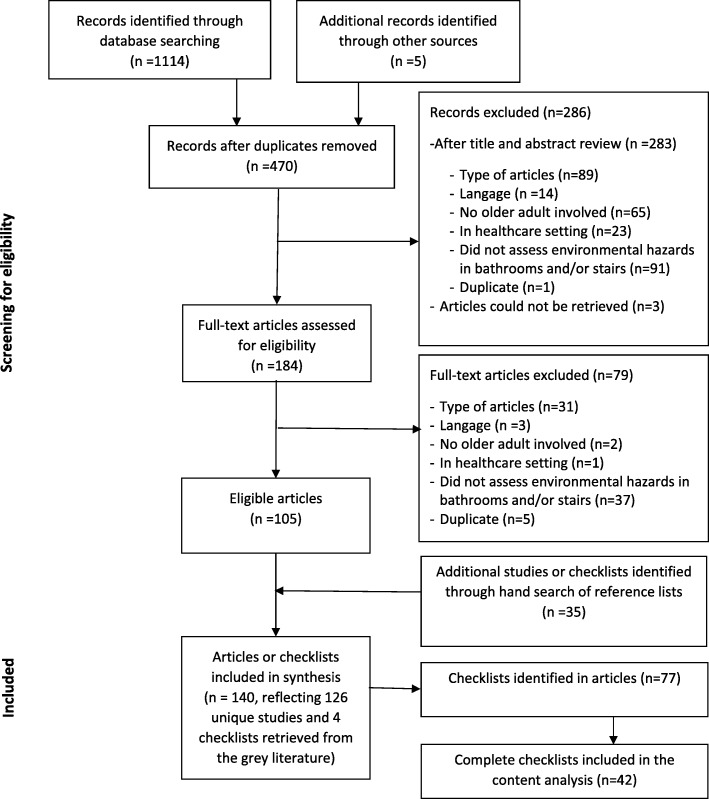
Screening process. Screening process for the scoping review on the assessment of environmental hazards for falls on stairs and in bathrooms

Figure 1 summarizes the two-stage process used to identify and select papers included in this review. The initial database searches yielded a total of 1114 articles. The search in HAPI and PsychTESTS yielded five additional articles, for a total of 1119 articles. All articles were entered in Zotero. Duplicates were removed, leaving 470 articles. First stage inclusion and exclusion criteria were pilot-tested and refined on a subset of 10 random titles and abstracts by the two authors. Titles and abstracts were then reviewed for stage one eligibility by two independent raters (first author and a research associate) and classified as eligible (*n* = 36), ineligible (*n* = 284) or unclear (*n* = 150). Any discrepancies in eligibility were discussed until a consensus was reached. Articles classified as eligible or unclear underwent full-text review by the first author. After full-text review, 105 articles were deemed eligible. An additional 35 eligible articles or checklists were identified through the hand search of reference lists.

A pool of 136 published articles [1, 5, 7, 8, 14–39, 44–64, 65–147] and 4 checklists from the grey literature [148–151] were included in the first stage of this scoping review. The 136 published articles represented 126 unique studies—nine studies had multiple citations.

From these documents, 42 unique checklists were identified and included in the second stage of this review [8, 15–17, 20–23, 34, 44, 49, 51, 53, 58, 66, 69, 72, 79, 82, 86, 87, 98, 102, 108, 110–112, 115, 121, 124, 126, 132, 135, 137, 139, 140, 144, 148–152].

### Data extraction

Each of the 140 articles or checklists identified during stage one was read thoroughly and all pertinent information extracted in Excel by the first author. Extraction was overseen by the second author. The key data fields extracted are detailed in Table 1. During stage two, the first author extracted details about which and how hazards were assessed in bathrooms or on stairs/steps (indoor and outdoor). Data extracted about studies described in multiple articles were combined. When checklists were described in multiple articles, data for the checklists were combined from all sources.

**Table 1:** Key data extraction fields for stage one and stage two publications

Stage and Fields	Description (and response options where applicable)
Stage 1	
Definition of environmental hazards	The definition of environmental hazards
Definition of falls	The definition of falls
Study objective	The objective of the study
Year	Year of the article publication; if more than one article originating from the same study, the year of the first article was selected
Country(ies)	Country(ies) where the study was conducted
Population	Specific characteristics of the study population
Assessors	Who assessed environmental hazards (occupational therapist; physiotherapist, nurse; researcher or research assistant; other professional; participants or family members)
Training or experience	Whether assessors were trained to use the checklist or experienced with home assessment (yes or no)
Quality of the training	Details about the training provided to assessors
Checklist name	The name of the specific checklist used
Psychometric properties	Validity or reliability of the checklist reported for the current study or another study using the same checklist.
Stage 2	
Environmental hazards analyzed/reported	All information provided about what and how hazards were evaluated in bathrooms and on stairs/steps (indoor and/or outdoor), and if and how the person-environment fit was assessed
Number of items	How many fall-related items were in the checklist (in total; in bathrooms; on indoor stairs/steps; and on outdoor stairs/steps)

An asterisk was inserted in database cells when information was not reported or not applicable. Questions that arose during the process about what data to extract were discussed until consensus was reached. The database is available upon request from the corresponding author.

### Data coding and analysis

#### Stage 1

We grouped studies or checklists into four main categories based on their objectives: 1- developed a checklist and/or tested its validity or reliability; 2- used a checklist to assess environmental hazards or the impact of environmental hazards on falls; 3- used a checklist in an intervention study and/or reported home modifications; 4- not applicable, checklist only. We dichotomized checklists according to reports of psychometric testing (those with versus those without reports of validity and/or reliability testing in current or previous studies) and assessor training (authors did or did not report training of assessors). Checklists were categorized according to whether they assessed solely fall-related hazards or whether they included non-fall-related environmental hazards. The former items were defined as “aspects of the physical environment, including objects, space and the elements in and about the house that pose a risk or danger of causing the person to fall” [56] (p. 171). Items considered unrelated to falls included fire hazards, medication misuse, and wandering.

Descriptive analyses were conducted in IBM SPSS Statistics for Windows (version 24.0, Armonk, NY). We examined whether or not reports of training assessors were associated with reports of developing checklist or testing its validity or reliability (yes/no) using a Pearson chi-square test. We tested the association between time (by 1-year and 5-year period) and the proportion of studies using checklists with prior psychometric testing using Spearman correlations. *P* values < 0.05 were considered significant.

#### Stage 2

Detailed information was extracted about how hazards were evaluated in bathrooms and on stairs/steps (indoor and/or outdoor), and if and how person-environment fit was assessed. This data was then content analyzed [43, 153] using two sets of categorical descriptors. First, we rated hazards as structural or non-structural. We defined structural hazards as environmental features that were anchored in walls or on floors (e.g., grab bars affixed to wall, handrails on stairs) or were features of building construction (e.g. stair geometry). We defined non-structural hazards as environmental features that were not anchored in walls or on floors (e.g., presence of bathmats, cluttered stairs). Second, we rated assessment criteria as objective or subjective. We defined objective criteria as defined physical properties not involving personal judgment (e.g. presence of handrail, tread length, lumens of light on stairs). We defined subjective criteria as undefined descriptors requiring the individual judgement of the assessor (e.g., steep or narrow stairs/steps, sturdy handrails or grab bars, slippery surface). Using these definitions, all items for the three locations of hazards (bathrooms, indoor stairs/steps and outdoor stairs/steps) were independently rated by the authors using the two sets of categories for increased internal reliability. Any discrepancies in ratings were discussed until a consensus was reached.

## Results

### Stage 1

#### Definitions of environmental hazards

Only 22 studies (17%) provided a definition for the term environmental hazards, and there was considerable variation in these definitions across studies. Most authors who defined hazards, described them by giving examples such as, “features of the home environment such as loose rugs, floor clutter, and poor lighting” [19] (p. 2) or “environmental features such as poor lighting, lack of handrails on staircases, objects in pathways, and slippery rugs” [25] (p. 16). The most comprehensive definition provided was “home fall hazards are aspects of the physical environment, including objects, space and the elements in and about the house that pose a risk or danger of causing the person to fall and, therefore, risk injury” [56] (p. 171).

#### Geographic location and objectives of studies

The 126 eligible studies and 4 checklists assessed hazards in 25 countries (Additional file 2: Table S1), with the leading sites being USA (*n* = 43, 33%), Australia (*n* = 17, 13%) and Canada (*n* = 13, 10%). Most studies had been undertaken in higher-income countries (*n* = 112, 86%).

Forty-one per cent (*n* = 52, 41%) of publications described used a checklist to assess environmental hazards and/or their impact on falls. Another 36% (*n* = 45, 36%) of publications presented intervention studies that aimed to prevent falls by reducing home environmental hazards. One quarter of studies (*n* = 32, 25%) reported the development of a checklist or tested its validity or reliability. Six studies were classified simultaneously in two of the above categories (*n* = 6, 5%), and six additional entries were categorized as solely the environmental hazard checklist (n = 6, 5%).

#### Checklist used

Seventy-seven different checklists were reported, with just one fourth (*n* = 19, 25%) used in two or more studies (see Table 2). Five checklists (6%) were used in at least five studies (Additional file 3: Table S2), the Westmead Home Safety Assessment (WeHSA, *n* = 10) [24, 48, 55–58, 61, 68, 136, 145], Minimum Data Set–Home Care instrument (MDS-HC; *n* = 7) [52, 54, 74, 99, 106, 112, 147], Tideiksaar et al. checklist (n = 7) [5, 14, 70, 94, 100, 129, 140], Home Falls and Accidents Screening Tool (HOME FAST; *n* = 6) [27, 101–103, 110, 122], and Housing Enabler Instrument (*n* = 5) [80, 83, 84, 118, 154]. A majority of studies (*n* = 57, 74%) used “in house” questionnaires. Only three checklists, the MDS-HC, Housing Enabler and Housing Enabler-screening tool had been used in cross-country studies [49, 80, 83, 84, 112].

**Table 2:** Environmental hazard checklists included in the content analysis

Checklist	Author	Year	Countries where checklists have been used	Total #of fall-related items in checklist	Psychometric data reported (Y/N)	Bathrooms				Indoor stairs/steps				Outdoor stairs/steps			
						#of fall-related items in bathrooms	> 50% of items are subjective (Y/N)	> 50 of items are non-structural hazards (Y/N)	Assessed grab bars (Y/N)	#of fall-related items on indoor stairs/steps	> 50% of items are subjective (Y/N)	> 50 of items are non-structural hazards (Y/N)	Assessed handrails (Y/N)	#of fall-related items on outdoor stairs/steps	> 50% of items are subjective (Y/N)	> 50 of items are non-structural hazards (Y/N)	Assessed handrails (Y/N)
Carter et al.	Carter et al. [51]	1997	Australia	99	Y	24	N	Y	Y	16	Y	N	Y	2	Y	Y	N
CDC Home checklist	CDC [148]	2005	USA, Singapore	25	N	6	Y	Y	N	7	N	N	Y	7	N	N	Y
Cougar^1^	Fisher et al. [71]	2006	USA	56	Y	5	Y	Y	Y	5	Y	N	Y	1	Y	Y	N
Edwards & Jones	Edwards & Jones [66]	1998	South Wales	14	N	5	N	Y	Y	1	N	N	Y	0	a	a	a
Environmental Safety Checklist	Huang et al. [16]	2005	China	31	Y	5	Y	Y	Y	4	Y	Y	Y	1	a	a	a
Evci et al.	Evci et al. [69]	2006	Turkey	15	N	6	N	N	Y	5	N	N	Y	0	a	a	a
Greene et al.	Greene et al. [79]	2009	USA	15	N	2	N	N	Y	4	N	N	Y	4	N	N	N
Healthy housing index	Keall et al. [90]	2008	New Zealand	26	N	3	Y	N		12	Y	Y		9	Y	Y	
HEAP	Gitlin et al. [15]	2002	USA	69	Y	10	N	Y	Y	6	Y	Y	Y	6	Y	N	Y
HEAVI	Yonge et al. [22]	2017	USA	127	Y	15	N	Y	Y	17	N	N	Y	19	N	N	Y
HEROS Environmental Safety Check	Sadasivam et al. [124]	2014	USA	5	Y	6	Y	Y	Y	0	a	a	a	0	a	a	a
Home Environment Survey	Rodriguez et al. [121]	1995	USA	17	Y	4	N	N	Y	Can’t tell	a	a	a	Can’t tell	a	a	a
HOME FAST	Mackenzie et al. [102]	2000	Australia, Scotland, Malaysia, England	25	Y	6	Y	Y	Y	4	Y	N	N	1	Y	N	Y
HOME FAST – Self-Report Assessment	Mehraban et al. [110]	2011	Australia	87	Y	7	Y	Y	Y	11	Y	N	Y	3	Y	N	Y
Home fall hazard assessment	You et al. (2004) [23]	2004	China	60	Y	16	Y	Y		10	Y	Y		0	a	a	
Home-screen safe	Johnson et al. [86]	2001	Australia	10	N	2	Y	Y	N	Can’t tell	a	a	a	Can’t tell	a	a	a
Housing Enabler^2^	Iwarsson & Björn [149]	2010	Sweden	188	Y	13	N	N	Y	11	N	N	Y	11	N	N	Y
Housing Enabler screening tool	Carlsson et al. [49]	2009	Sweden, Germany, Latvia, Hungary, Denmark, Finland, Iceland, England	61	Y	10	Y	Y	Y	6	Y	N	Y	6	Y	N	Y
Isbener et al.	Isbener et al. [82]	1998	USA	21	N	0	a	a	a	10	Y	N	Y	2	N	N	N
Kamei et al.	Kamei et al. [87]	2015		33	N	7	Y	Y	Y	Can’t tell	Y	N	Y	Can’t tell	a	a	a
Kellogg international work group	Kellogg international work group [139]	1987	Canada, USA	40	N	4	Y	Y	Y	6	Y	N	Y	0	a	a	a
Lan et al.	Lan et al. [17]	2009		6	N	3	Y	Y	Y	0	a	a	a	1	Y	N	Y
Lim & Sung	Lim & Sung [126]	2012	Korea	3	N	1	N	Y	N	0	a	a	a	0	a	a	a
MAHC-10 Fall Checklist	Bamgbade & Dearmon [44]	2016	USA	1	N	< 1	a	a	N	Can’t tell	a	a	a	Can’t tell	a	a	a
Marshall et al.	Marshall et al. [108]	2005	USA	10	N	2	N	N	Y	1.5	N	N	Y	1.5	N	N	Y
McLean & Lord	McLean & Lord [34]	1996	Australia	15	N	4	N	Y	Y	0	a	a	a	2	N	N	Y
MDS-HC	Morris et al. [112]	1997	Canada, Hong Kong, USA, Korea, Italy, Australia, Czech Republic, Japan	8	Y	1	Y	Y	Y	1	Y	N	N	1	Y	Y	N
Morgan et al.	Morgan et al. [111]	2005		73	Y	13	Y	Y	Y	2	Y	N	Y	4	N	N	Y
Nevit et al.	Nevit et al. [7] reported in Northridge et al. [115]	1898	USA	22	Y	8	Y	Y	Y	0	a	a	a	0	a	a	a
Safe living guide	Public Health Agency of Canada [151]	2015	Canada	34	Y	9	Y	Y	Y	6	Y	Y	Y	2	Y	N	Y
SAFER-HOME	Chiu & Oliver [53]	2006	Canada, USA	17	Y	7	Y	Y	Y	2	Y	N	Y	0	a	a	N
SAFER Tool	Letts et al. [98]	1998	Canada	17	Y	5	Y	Y	Y	2	Y	N	Y	0	a	a	N
Sattin et al.	Sattin et al. [8]	1998	USA	10	Y	3	N	Y	Y	0	a	a	a	0	a	a	a
Sophonratanapokin et al.	Sophonratanapokin et al. [132]	2012	Thailand	9	N	4	N	N	Y	2	N	N	Y	1	N	N	N
Stalenhoef et al.	Stalenhoef et al. [135]	1998	Netherlands	116	Y	17	Y	Y	Y	11	Y	N	Y	0	a	a	a
Stevens et al.	Stevens et al. [137]	2001	Australia	14	N	8	Y	Y	N	2	N	N	N	0	a	a	a
Tanner	Tanner [20]	2003	USA	20	N	2	N	N	Y	Can’t tell	Y	N	Y	Can’t tell	a	a	a
Tideiksaar et al.	Tideiksaar et al. [140]	1987	Canada, England, USA	37	Y	6	Y	N	Y	4	Y	N	Y	5	Y	N	Y
Vladutiu et al.	Vladutiu et al. [144]	2012	USA	6	N	2	N	N	Y	3	N	N	Y	1	N	N	N
WeSHA	Clemson et al. [58]	1992	Australia, England, New Zealand, USA	72	Y	10	Y	Y	Y	7	Y	N	Y	5	Y	N	Y
Wyman et al.	Wyman et al. [21]	2007	USA	37	Y	12	Y	Y	Y	7	Y	N	Y	0	a	a	a
Zhang et al.	Zhang et al. [152]	2016	China	30	Y	8	Y	Y	Y	6	Y	N	Y	1	Y	N	N

*Y*=Yes, *N*=No

*CDC*: Center for disease control

*HEAP*: Home Environmental Assessment Protocol

*HEAVI*: Home Environment Assessment for the Visually Impaired

*HOME FAST*: Home Falls and Accidents Screening Tool

*MAHC-10*: Missouri Alliance for Home Care-10

*MDS-HC*: Minimum Data Set-Home Care

*SAFER-HOME*: Safety Assessment of Function and the Environment for Rehabilitation–Health Outcome Measurement and Evaluation

*SAFER Tool*: Safety Assessment of Function and the Environment for Rehabilitation

*WeHSA*: Westmead Home Safety Assessment

^a^Location not assessed

^1^ Version 1.0 and 2.0;

^2^ Original, revised and Nordic

#### Psychometric properties of checklists

**Fig. 2:**
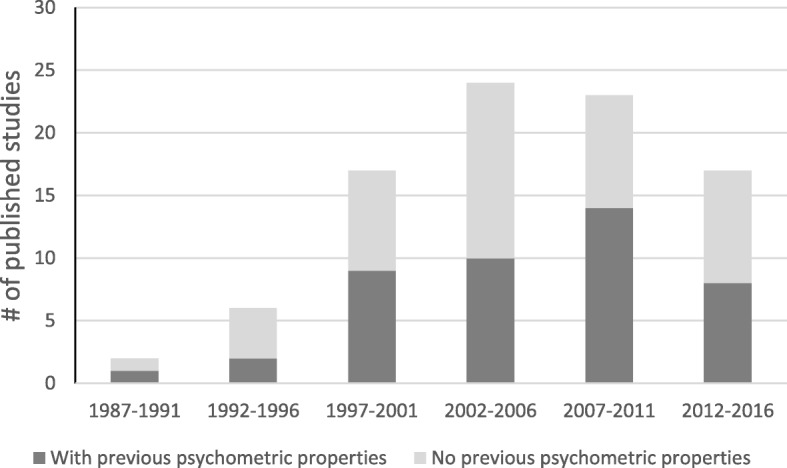
Number of articles published according to whether the checklist used had prior psychometric testing (*n* = 96)

Most studies summed up hazardous items into an overall safety score. There was little discussion of the clinical appropriateness of this approach. Studies varied markedly in terms of the psychometric data presented. Some authors reported criterion validity [49, 62, 110, 112, 120, 124], others reported content validity [56, 81, 82, 91, 97, 103] or predictive validity [27]. Only two authors reported sensitivity and specificity of checklists items [62, 103]. Thirty studies reported inter-rater reliability [8, 14, 15, 19, 23, 51, 54, 57–59, 72, 77, 80, 81, 84, 85, 98, 103, 106, 110–112, 115, 120–123, 133, 135, 152]; fewer reported test-retest reliability [36, 81, 98, 122] or internal consistency [53, 81, 86, 97]. The inter-rater reliability of checklists, when used by professional and lay older adult pairs, was reported in four studies; three showed that professionals identified more hazards than lay older adults [23, 111, 133] and one showed that lay older adults reported more of some hazards, while professionals reported more of other hazards [110]. Further, the reliability of items on a checklist was often reported as excellent for some but poor for others [8, 19, 58, 80, 103, 110, 111, 115, 121]. Two authors noted that objective items had a higher reliability coefficient than subjective items [19, 80]. Interestingly, no time trend was observed in the proportion of studies using checklists with prior psychometric testing versus checklists without this prior testing (see Fig. 2).

#### Study populations

About half of the studies (*n* = 73, 57%) drew their sample from the general population. The remainder targeted populations at a higher risk of falls such as individuals who had fallen in the previous year; frail individuals; or individuals with mental or visual impairments.

Almost no studies adapted the type of specific home hazards assessed to the specific needs of participants. There were two exceptions. The HEAVI was developed for visually impaired individuals and focusses on related environmental features such as lighting and visual cues [19]. The HEAP was developed for individuals with dementia and includes an assessment of pressure gates at the top and bottom of stairways [15, 78].

#### Who completed the assessment

Among the studies that assessed environmental hazards (*n* = 122), evaluations were conducted by occupational therapists (*n* = 45, 37%), nurses (*n* = 28, 23%), researchers or research assistants (*n* = 20, 16%), the participant or a family member (*n* = 18, 15%), other professionals (*n* = 16, 13%; e.g.; physicians, home inspectors, house retailers), or physiotherapists (*n* = 7, 6%). In 17 studies (14%), two or more types of assessors conducted assessments. Assessors were not described in 7 studies (6%). Forty-nine (40%) studies specified that assessors had been trained or had experience in home assessment, and two studies (2%) mentioned that the checklist used does not require prior training in home evaluation or modification [18, 21]. The rest of the studies (*n* = 69, 57%) provided no details about training. The assessments in 22 (32%) of these latter studies were conducted by occupational therapists. When described, training approaches varied in duration (one-hour to one-week workshop) and format (theoretical lectures, video of home assessment or practical sessions using the checklist in real/mock homes). Studies that described training assessors were more likely to report developing a checklist or testing its validity or reliability (X^2^ = 15.840, df = 1, *p* < 0.0001, Table 3).

**Table 3:** Association between training assessors and developing a checklist or testing its validity or reliability

	Report developing a checklist or testing its validity or reliability		
Report training assessors	Yes (*n* = 67)	No (*n* = 53)	p*
Yes	56.7%	20.8%	< 0.0001
No	43.3%	79.2%	

*Chi-square analysis

### Stage 2

Most checklists assessed solely fall-related environmental hazards; a quarter of them (*n* = 10) were imbedded in a checklist designed to also capture non-fall related hazards. As shown in Tables 2 and 4, checklists varied greatly in their length and in the number of bathroom and stair items assessed. Checklists differed with respect to what rooms were assessed (e.g. all bathrooms or bathroom most often used), whether or not outdoor hazards were assessed, and how responses were coded. Some hazards were assessed using dichotomous response categories (e.g.; present/absent); others were coded as continuous variables (e.g.; number of stairs/steps).

**Table 4:** Bathroom and stair locations included in checklists and range and average number of items evaluated (*n* = 42 checklists)

Location of assessment items	Checklists including ≥1 items n (%)	Range for number of items	Average (SD) number of items
Bathrooms	39 (92.9%)	1–24	7.2 (5.1)
Indoor stairs/steps	33 (78.5%)	1–17	6.0 (4.2)
Outdoor stairs/steps	23 (54.7%)	1–19	4.2 (4.2)
All locations	42 (100%)	1–188	38.1 (38.8)

Range and average are shown only for tools including 1 or more assessment item in location

#### Person-environment fit

Most checklists did not assess person-environment fit. There were a few exceptions [15, 34, 53, 58, 69, 83, 87, 98, 102, 110, 140]. Examples of items that assessed person-environment fit included either participants’ self-reports or assessors’ observations of difficulties (or lack or thereof) walking from room to room, over different floor surfaces; climbing and descending stairs/steps; transferring from beds, chairs, and toilets; and getting in and out of bathtubs or showers. Notably, the Housing Enabler Instrument [83] assesses the environment and older adults’ functional limitations separately. Uniquely, these authors provide an analytic matrix and a software program to examine the gap between the environment and the person’s limitations.

#### Summary of key findings related to bathrooms

Thirty-nine checklists assessed bathrooms. Few checklists indicated which bathroom to assess when there were more than one in the home. As shown in Table 5, a majority of checklists (*n* = 25, 63%) used mostly subjective items to assess hazards in bathrooms. For instance, they assessed an “awkward toilet seat”, or “slippery floor”. Similarly, over three quarters of checklists (*n* = 30, 77%) assessed primarily non-structural hazards such as non-skid mats, abrasive strips in the bath or shower, or objects on the bathroom floor. The most frequently assessed structural hazard was the absence of grab bars.

**Table 5:** Summary of findings on how environmental hazards are assessed in bathrooms (*n* = 39 checklists)

Characteristics of items assessed on checklists	n (%)
Overall	
> 50% of items are subjective	25 (63%)
> 50% of items are non-structural hazards	30 (77%)
Frequently assessed hazards	
Assessed the presence of throw rugs and/or if they were well anchored to the floor (non-structural hazards)	20 (51%)
Assessed the lack of a non-skid mat or strips in the tub and/or shower (non-structural hazards)	22 (56%)
Assessed the absence of grab bars (structural hazards)	35 (90%)

##### Grab bars

Although most checklists assessed grab bars in bathrooms, there was a lot of variation as to where (in the bathtub, shower and/or next to the toilet) and how they were assessed. For instance, in some checklists, grab bars were assessed with a single item and a bathroom would need to have grab bars in three locations (toilet, bath, shower) not to be hazardous, while in others, grab bars were also assessed with a single item but the presence of only one grab bar was enough to code the item as having the grab bar present. In other studies, each location was assessed separately and one, two or three items were listed in the checklist accordingly. Two checklists coded the lack of grab bars as a hazard only if the person needed them [23, 115], one coded using a grab bar as a hazard [20], and another described grab bars as assistive devices and did not consider their absence to be an environmental hazard [108].

None of the checklists distinguished between diagonal, horizontal or vertical grab bars in the tub/shower; or documented where they were situated (e.g. side wall and/or back wall). Four checklists assessed grab bar placement; one had objective height measures [149], whereas others relied on subjective criteria such as “properly installed” [71], “properly placed” [151], or “can be reached without leaning enough to lose balance” [102]. Four checklists assessed if grab bars were sturdy or well anchored to walls [16, 139, 140, 151]. Illustrations of grab bars in another checklist included two types that were not fixed to a wall [111]. Only three checklists provided a definition of grab bars or specified that towel racks are not grab bars [102, 111, 121].

#### Summary of key findings related to stairs/steps

Thirty-nine checklists included items on stairs and/or steps. Most (*n* = 22, 63%) assessed both indoor and outdoor stairs/steps, while eleven (31%) assessed only indoor stairs/steps and two assessed only outdoor stairs/steps. The location of stairs/steps (whether indoors or outdoors) was not differentiated in four of the checklists (10%). Very few checklists assessed the number of stairs/steps or staircases in the home.

##### Indoor stairs/steps

Among the 33 checklists that assessed indoor stairs/steps, twenty-six (79%) assessed features of stairs/steps not related to handrails (see Table 6). Most checklists (*n* = 23, 70%) used a majority of subjective items (e.g. stairs/steps in need of repair, sloping or broken steps, stairs too steep) and most (*n* = 30, 91%) included a majority of structural items. Yet, most structural features assessed were not related to the construction geometry of stairs/steps (e.g., height of riser, tread width).

**Table 6:** Summary of findings on how environmental hazards are assessed in stairs/steps

Characteristics of items assessed on checklists	n (%)
Indoor stairs/steps (*n* = 33 checklists)	
> 50% of items are subjective	23 (70%)
> 50% of items are non-structural hazards	3 (9%)
Assessed handrails absence or features (structural hazard)	30 (91%)
Outdoor stairs/steps (n = 23 checklists)	
> 50% of items are subjective	13 (57%)
> 50% of items are non-structural hazards	3 (13%)
Assessed handrails absence or features (structural hazard)	15 (65%)

##### Indoor handrails

Handrails were the most commonly assessed structural features of stairs/steps (n = 30, 91%). Eight checklists (27%) solely assessed if handrails were present; the others assessed specific features of handrails: sturdiness (*n* = 18, 60%); height, length and/or if they were continuous (*n* = 13, 43%); and diameter or ease of grip (*n* = 7, 23%). There was also variability in the number of handrails that needed to be present to code stairs/steps as not hazardous. For the majority (*n* = 24, 80%) the presence of only one handrail resulted in this categorization, while for six checklists (*n* = 6, 20%), two handrails had to be present for this categorization.

##### Outdoor stairs/steps

There were fewer items and fewer features assessed for outdoor than indoor stairs/steps. Of the 23 checklists that assessed outdoor stairs/steps, eight (35%) assessed some features of stairs/steps other than handrails. Assessment criteria were predominantly subjective in most of these checklists (*n* = 13, 57%). Most checklists assessed structural hazards (*n* = 20, 87%). Yet, similarly to indoor stairs/steps, the features assessed were not related to their construction geometry.

##### Outdoor handrails

Handrails were assessed in 15 (65%) checklists. Almost all of these checklists assessed at least one specific feature of handrails (n = 13, 87%): sturdiness (*n* = 7, 50%); height, length and/or if they were continuous (*n* = 5, 31%); and diameter or ease of grip (n = 2, 13%). Similarly to the assessment of indoor handrails, 87% (n = 13) of checklists required the presence of only one handrail to code outdoor stairs/steps as not hazardous.

## Discussion

This is the first scoping review to examine the characteristics of environmental hazards checklists. Given the pervasive presence of environmental hazards in homes and their causal relationship with falls and independent functional mobility among the older adults, examining the status and quality of such checklists is imperative.

Despite over three decades of research in this field, there are still no widely used environmental assessment checklists. There is a lot of variability among checklists in terms of the number of items, which parts of the home were assessed, and among those assessing bathrooms and stairs/steps whether checklists emphasized structural or non-structural features or used primarily objective or subjective criteria for assessments. The lack of standardized assessment items in checklists severely limits cross-study comparisons [58]. In 2003, Gitlin concluded that there was a “lack of psychometrically sound measures” to assess home environments and that most assessment methods used were study-specific with unknown reliability and validity [155]. Our review indicates that this conclusion still largely holds. Developing “gold standard” environmental hazards checklists with known psychometric properties is critical to advance the field and inform fall-related prevention practices. This requires the development of a consensus definition of environmental hazards [58], and the identification of priority structural and non-structural attributes of safe bathrooms and safe stairs/steps. There is substantial data available from ergonomic studies to support this prioritization. Furthermore, statistical modeling of the relationship between checklist items and falls would help establish the predictive validity of checklist items, determine if it is clinically appropriate to sum all items into an overall hazard score, and identify priority objective measures for inclusion in abbreviated checklists.

We found limited descriptions of training approaches used and a lack of information on whether or not assessors were trained to use checklists. We recognize that training is costly, but agree with authors who have suggested that training is essential to achieve consistent assessments among raters [57]. For instance, interviewers have been shown to incorrectly identify towel racks as grab bars [8], highlighting the need to train them and to provide definitions of hazards. We also think that scaling-up the use of robust environmental hazard assessment checklists is important; their reach could be extended by training lay people to conduct assessments, and reducing the number of items on hazard checklists.

Given the disproportionately high rate of injurious falls that occur on stairs/steps and in bathrooms [10–12], it was surprising to us that checklists did not always include an assessment of these locations and that outdoor stairs/steps were so infrequently included. Outdoor stairs/steps often comprise part of older adults’ walking paths (Edwards & Dulai, under review); affect the visitability of a home; and may be more prone to hazardous characteristics since they may not be covered by building code legislation. In our view, comprehensive environmental hazard checklists need to assess both indoor and outdoor home environments.

Most of the authors describing environmental hazard checklists seemed to conceptualize the environment as an independent static entity, ignoring how older adults interact with their environment or the degree of their exposure [155, 156]. Ideally, checklists that assess person-environment fit and/or dynamic variability of the environment would be used alongside standard checklists, providing more insights on how older adults navigate their home environment in ways that either reduce or increase their risk of falls [157]. For example, checklists should assess whether older adults use stair handrails to compensate for poor balance or use a toilet or bathtub grab bar to aid transfers. Checklists should also contain items and directions pertaining to assessing the dynamic and variable nature of some environmental hazards (e.g. outdoor stairs/steps that were dry versus covered in ice or snow, friction coefficient of wet versus dry bathroom floor, combinations of natural and artificial lighting on stairs/steps that changed at different times of the day) [56].

There has been a tendency to define the problem of environmental risk modification as an individual behaviour change problem rather than as an environmental issue that requires a multi-level and inter-sectoral approach such as building code legislation and regulated universal design [158]. This behavioural emphasis may in part, explain the emphasis on subjective and non-structural items that was evident in checklists that assessed bathrooms and stairs/steps. In the longer-term, policy interventions, are likely to be more effective than behavioural interventions in facilitating some environmental modifications, such as safer stair geometry and universal access to grab bars for toilets, showers and bathtubs [159, 160]. It is imperative that we identify those constellations of hazards that are priorities and best tackled through policy change. This requires cumulative knowledge about the prevalence of structural environmental hazards and their relationship to falls. The inclusion of consistent, objectively-assessed, structural items in environmental hazard checklists could help address this knowledge gap.

### Limitations

This review has several limitations. First, we focussed on hazards related to bathrooms and stairs/steps. This may have resulted in the exclusion of a few checklists assessing solely other parts of homes. Second, we did not attempt to access unpublished training manuals for checklists, which may include descriptions of items that would have led us to categorize them as objective rather than subjective. However, most studies did not mention training their assessors or having a training manual, so it seems unlikely that this would have substantially shifted our results. Third, it was sometimes hard to categorize items as structural or non-structural, or as objective or subjective due to the limited descriptors of hazards contained in many checklists. For instance, “dim lightning” could be caused by a lack of proper ceiling light fixtures (structural) or by a burned-out light bulb (non-structural). To improve reliability, both authors independently rated the environmental hazard items on checklists and discussed discrepant results until a consensus emerged. However, it might have been more rigorous to involve an independent rater in this process. Lastly, we did not judge the appropriateness of objective criteria used to evaluate hazards. We did observe that objective criteria were inconsistent across checklists. In the future, an assessment of objective criteria should include a quality assessment against standards such as those suggested in ergonomic studies or those used in existing building code legislation.

## Conclusion

The lack of standard definitions and consistent objective criteria for assessing environmental hazards for falls is limiting meaningful cross-study comparisons and slowing advances in this field. This gap may partly explain conflicting results regarding the effectiveness of interventions targeting home environmental hazards (in particular those involving bathrooms and stairs/steps) to prevent falls among older adults. This field of research would be improved with standardized environmental hazard checklists containing objective criteria to assess structural hazards. To inform population health interventions aimed at preventing falls, such as building code regulations or municipal housing by-laws, it is essential to include objectively-assessed, structural hazards in environmental checklists.

## Additional files

Additional file 1: Detailed search strategy (DOCX 19 kb)

Additional file 2: **Table S1.** Environmental hazard checklists used by country (XLSX 15 kb)

Additional file 3: **Table S2.** Characteristics of assessment tools used in five studies or more (XLSX 17 kb)

## Abbreviations

CDC: Center for Disease Control
CINAHL: Cumulative Index to Nursing and Allied Health Literature
HAPI: Health and Psychosocial Instruments
HEAP: Home Environmental Assessment Protocol
HEAVI: Home Environment Assessment for the Visually Impaired
HOME FAST: Home Falls and Accidents Screening Tool
MAHC-10: Missouri Alliance for Home Care-10
MDS-HC: Minimum Data Set-Home Care
MeSH: Medical Subject Headings
PRISMA: Preferred Reporting Items for Systematic Reviews and Meta-Analyses
WeHSA: Westmead Home Safety Assessment
