# Supplementary material for: A need to improve the assessment of environmental hazards for falls on stairs and in bathrooms: results of a scoping review
Source: BMC Geriatr. 2018 Nov 9;18:272. doi: 10.1186/s12877-018-0958-1 (PMC6234792; doi:10.1186/s12877-018-0958-1)
Supplement: Supplementary file 1 — Detailed search strategy (DOCX 19 kb) [file 12877_2018_958_MOESM1_ESM.docx]

**Detailed search strategy**

**Web of Science**

| #4 | #3 AND #2 AND #1 |
| --- | --- |
| #3 | **TOPIC:** (home) *OR* **TOPIC:** (house) *OR* **TOPIC:** (housing) *OR* **TOPIC:** (dwelling) |
| #2 | **TOPIC:** (environment* NEAR/3 hazard*) *OR* **TOPIC:** (home NEAR/3 hazard*) |
| #1 | **TOPIC:** (fall) *OR* **TOPIC:** (falls) *OR* **TOPIC:** (falling) |

**Embase**

| 1. fall risk/ or fall risk assessment/ or falling/ |  |
| --- | --- |
| 2. fall*.mp. [mp=title, abstract, heading word, drug trade name, original title, device manufacturer, drug manufacturer, device trade name, keyword, floating subheading word] |  |
| 3. 1 or 2 |  |
| 4. (home adj3 hazard*).mp. [mp=title, abstract, heading word, drug trade name, original title, device manufacturer, drug manufacturer, device trade name, keyword, floating subheading word] |  |
| 5. (environment* adj3 hazard*).mp. [mp=title, abstract, heading word, drug trade name, original title, device manufacturer, drug manufacturer, device trade name, keyword, floating subheading word] |  |
| 6. 4 or 5 |  |
| 7. housing/ |  |
| 8. home for the aged/ |  |
| 9. home.mp. [mp=title, abstract, heading word, drug trade name, original title, device manufacturer, drug manufacturer, device trade name, keyword, floating subheading word] |  |
| 10. housing.mp. [mp=title, abstract, heading word, drug trade name, original title, device manufacturer, drug manufacturer, device trade name, keyword, floating subheading word] |  |
| 11. dwelling.mp. [mp=title, abstract, heading word, drug trade name, original title, device manufacturer, drug manufacturer, device trade name, keyword, floating subheading word] |  |
| 12. 7 or 8 or 9 or 10 or 11 |  |
| 13. 3 and 6 and 12 |  |

**Medline**

| 1. accidental falls/ or accidents, home/ |  |
| --- | --- |
| 2. fall.mp. [mp=title, acronym, descriptors, measure descriptors, sample descriptors, abstract, source] |  |
| 3. 1 or 2 |  |
| 4. (home adj3 hazard*).mp. [mp=title, acronym, descriptors, measure descriptors, sample descriptors, abstract, source] |  |
| 5. (environment* adj3 hazard*).mp. [mp=title, acronym, descriptors, measure descriptors, sample descriptors, abstract, source] |  |
| 6. 4 or 5 |  |
| 7. 3 and 6 |  |
| 8. Housing/ or Public Housing/ or Housing for the Elderly/ |  |
| 9. home.mp. [mp=title, acronym, descriptors, measure descriptors, sample descriptors, abstract, source] |  |
| 10. dwelling.mp. [mp=title, acronym, descriptors, measure descriptors, sample descriptors, abstract, source] |  |
| 11. 8 or 9 or 10 |  |
| 12. 7 and 11 |  |
| 13. Aged/ |  |
| 14. senior.mp. [mp=title, acronym, descriptors, measure descriptors, sample descriptors, abstract, source] |  |
| 15. aged.mp. [mp=title, acronym, descriptors, measure descriptors, sample descriptors, abstract, source] |  |
| 16. elderly.mp. [mp=title, acronym, descriptors, measure descriptors, sample descriptors, abstract, source] |  |
| 17. 13 or 14 or 15 or 16 |  |
| 18. 12 and 17 |  |

**Scopus**

TITLE-ABS-KEY ( ( fall* )  AND  ( ( home )  OR  ( housing )  OR  ( dwelling ) )  AND

( ( environment*  PRE/3  hazard* )  OR  ( home*  PRE/3  hazard* )  OR  ( fall  PRE/3  hazard* ) ) )

**AgeLine**

| S10 | S5 AND S9 |
| --- | --- |
| S9 | S6 OR S7 OR S8 |
| S8 | home |
| S7 | dwelling |
| S6 | housing |
| S5 | S1 AND S4 |
| S4 | S2 OR S3 |
| S3 | environment* N3 hazard* |
| S2 | home N3 hazard* |
| S1 | fall* |

**CINAHL**

| S23 | S17 AND S22 |
| --- | --- |
| S22 | S18 OR S19 OR S20 OR S21 |
| S21 | housing |
| S20 | dwelling |
| S19 | home |
| S18 | (MH "Housing") OR (MH "Public Housing") OR (MH "Housing for the Elderly") OR (MH "Home Visits") |
| S17 | S13 AND S16 |
| S16 | S14 OR S15 |
| S15 | environment* N3 hazard* |
| S14 | home N3 hazard* |
| S13 | S11 OR S12 |
| S12 | fall* |
| S11 | (MH "Accidental Falls") OR (MH "Accidents, Home") |
| S10 | S5 AND S9 |
| S9 | S6 OR S7 OR S8 |
| S8 | home |
| S7 | dwelling |
| S6 | housing |
| S5 | S1 AND S4 |
| S4 | S2 OR S3 |
| S3 | environment* N3 hazard* |
| S2 | home N3 hazard* |
| S1 | fall* |
